# Dataset on Investigating the role of onsite learning in the optimisation of craft gang's productivity in the construction industry

**DOI:** 10.1016/j.dib.2017.09.073

**Published:** 2017-10-05

**Authors:** Rex Asibuodu Ugulu, Stephen Allen

**Affiliations:** School of Construction Economics and Management, the University of Witwatersrand, 1 Jan Smuts Avenue, Braamfontein, Johannesburg 2000, South Africa

**Keywords:** Onsite learning, Construction productivity, Learning curve theory, Blockwork and Craft gang's

## Abstract

The data presented in this article is an original data on “Investigating the role of onsite learning in the optimisation of craft gang's productivity in the construction industry”. This article describes the constraints influencing craft gang's productivity and the influence of onsite learning on the blockwork craft gang's productivity. It also presented the method of data collection, using a semi-structured interview and an observation method to collect data from construction organisations. We provided statistics on the top most important constraints affecting the craft gang's productivity using 3-D Bar charts. In addition, we computed the correlation coefficients and the regression model on the influence of onsite learning on craft gang's productivity using the man-hour as the dependent variable. The relationship between blockwork inputs and cycle numbers was determined at 5% significance level. Finally, we presented data information on the application of the learning curve theory using the unit straight-line model equations and computed the learning rate of the observed craft gang's blockwork repetitive work.

**Specifications Table**

**Table:** 

Subject area	*Economics, Construction Management, Project management, Management, Quantity surveying and Civil Engineering.*
More specific subject area	*Construction Project Management*
Type of data	*Table, Figures.*
How data was acquired	*Data was acquired by conducting a Semi-structure Interview and observation of the craft gang's in the observed project site.*
Data format	*Raw, filtered, analyzed.*
Experimental factors	*We make use of interview and observational data. Our sample was through purposeful.*
Experimental features	*Data on interview transcript, observed craft gang's man-hour labour productivity.*
Data source location	*Nigeria.*
Data accessibility	*The data are available with this article.*
Related research article	*The data is not related to a companion paper to any research article.*

**Value of the data**

• The presented data in Fig. 1, Fig. 2, Fig. 3 on the project-specific constraints influencing blockwork craft gang's productivity could inform further research on constraints influencing craft gang's productivity.

**Fig. 1:**
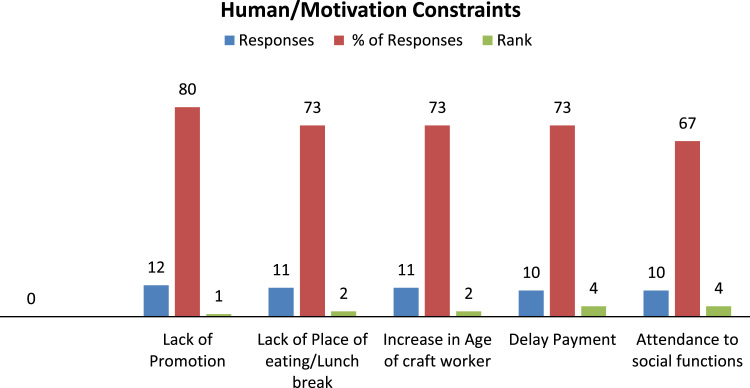
Top 5 human/motivation constraints.

**Fig. 2:**
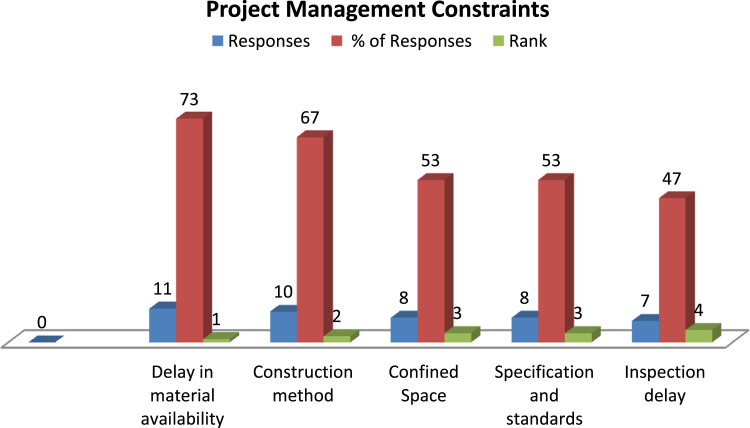
Top 5 project management constraints.

**Fig. 3:**
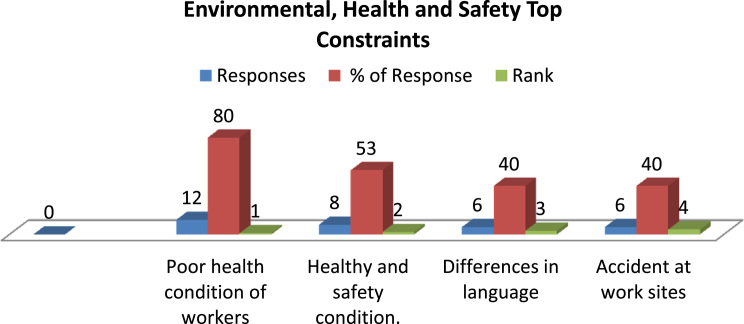
Top environmental, health and safety top constraints.
• Craft gangs learning rate productivity determine in Table 3 and Fig. 4 can stimulate further research on craft gang's productivity using U-block, solid walls and curve walls.

**Fig. 4:**
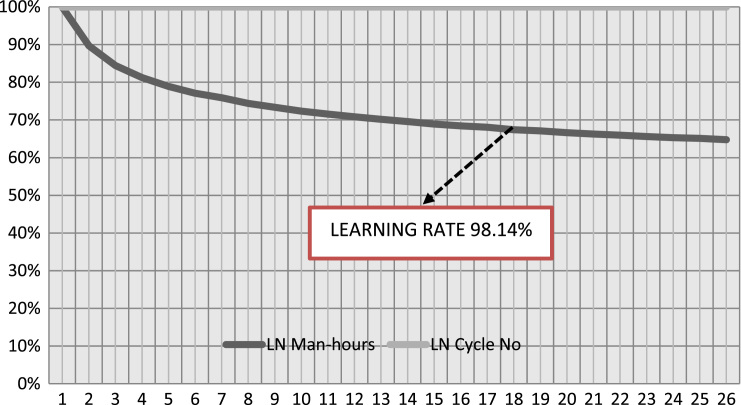
Relationship of blockwork craft gangs productivity and cycle numbers.
• The data on Fig. 4 and Table 3 are further evidence on the application of the learning curve theory to blockwork craft gang's.
• The data in this article could be useful to optimise further onsite craft gang's productivity within a project specific environment.

## 1 Data

In this article, first we presented three 3-D bar charts representing the top constraints influencing onsite craft gang's productivity (Fig. 1, Fig. 2, Fig. 3). The correlation coefficient table and the overall regression model between the productive input and its associated cycle number in Table 1, Table 2 was computed using simple linear regression technique. Table 3 present the learning rate of the observed repetitive work activity in the project.

**Table 1:** Regression correlation coefficient for blockwork craft gang's.

S/N	**LN Man-hours**	**LN Cycle No**	**C**	**D**	**E**	**F**	**G**	**H**	**I**	**J**	**K**	**L**	**M**	**N**	**O**	**P**
	Y	X	XY	X^2^	Y^2^	n∑XY	∑X∑Y	n∑X^2^	(∑x)^2^	n∑Y^2^	(∑Y)^2^	H-I	J-K	(L*M)^1/2	F-G	ϒ=O/N
1	6.10	–	0	–	37.2100	9579.295908	9591.644112	4209.966516	3754.35602	**24,505.59**	24504.7716	455.6104957	0.82	19.28154985	−12.348204	**−0.640415532**
2	6.04	0.6931	4.18632	0.4804	36.4816											
3	6.03	1.1098	6.69209	1.2317	36.3609											
4	6.01	1.3862	8.33106	1.9216	36.1201											
5	6.01	1.6094	9.67249	2.5902	36.1201											
6	6.03	1.7917	10.804	3.2102	36.3609											
7	6.12	1.9459	11.9089	3.7865	37.4544											
8	6.04	2.0794	12.5596	4.3239	36.4816											
9	6.05	2.1972	13.2931	4.8277	36.6025											
10	6.02	2.3025	13.8611	5.3015	36.2404											
11	6.04	2.3978	14.4827	5.7494	36.4816											
12	6.03	2.4849	14.9839	6.1747	36.3609											
13	6.04	2.5649	15.492	6.5787	36.4816											
14	6.04	2.6390	15.9396	6.9643	36.4816											
15	6.00	2.7080	16.248	7.3333	36.0000											
16	6.01	2.7725	16.6627	7.6868	36.1201											
17	6.04	2.8332	17.1125	8.0270	36.4816											
18	5.98	2.8903	17.284	8.3538	35.7604											
19	6.01	2.9444	17.6958	8.6695	36.1201											
20	5.98	2.9957	17.9143	8.9742	35.7604											
21	5.98	3.0445	18.2061	9.2690	35.7604											
22	5.99	3.0918	18.5199	9.5592	35.8801											
23	5.98	3.1355	18.7503	9.8314	35.7604											
24	5.98	3.1781	19.005	10.1003	35.7604											
25	6.01	3.2189	19.3456	10.3613	36.1201											
26	5.98	3.2581	19.4834	10.6152	35.7604											
**∑**	**156.5**	**61.2728**	**368.434**	**161.9218**	**942.5226**

**Table 2:** Regression Model for Blockwork Craft gang's.

S/N	LN Man-hours	LN Cycle No	C	D	E	F	G	H	I	J	K	M	N	O
	Y	X	XY	X^2^	Y^2^	n∑XY	∑X∑Y	n∑X^2^	(∑x)^2^	H-I	F-G	β=K/J	Βẋ	α=Y¯-βẊ
1	6.10	–	0	–	37.2100	9579.295908	9591.644112	4209.966516	3754.35602	455.6104957	−12.348204	−0.0271	−0.063871109	**6.08464**
2	6.04	0.6931	4.186324	0.4804	36.4816									
3	6.03	1.1098	6.692094	1.2317	36.3609									
4	6.01	1.3862	8.331062	1.9216	36.1201									
5	6.01	1.6094	9.672494	2.5902	36.1201									
6	6.03	1.7917	10.80395	3.2102	36.3609									
7	6.12	1.9459	11.90891	3.7865	37.4544									
8	6.04	2.0794	12.55958	4.3239	36.4816									
9	6.05	2.1972	13.29306	4.8277	36.6025									
10	6.02	2.3025	13.86105	5.3015	36.2404									
11	6.04	2.3978	14.48271	5.7494	36.4816									
12	6.03	2.4849	14.98395	6.1747	36.3609									
13	6.04	2.5649	15.492	6.5787	36.4816									
14	6.04	2.6390	15.93956	6.9643	36.4816									
15	6.00	2.7080	16.248	7.3333	36.0000									
16	6.01	2.7725	16.66273	7.6868	36.1201									
17	6.04	2.8332	17.11253	8.0270	36.4816									
18	5.98	2.8903	17.28399	8.3538	35.7604									
19	6.01	2.9444	17.69584	8.6695	36.1201									
20	5.98	2.9957	17.91429	8.9742	35.7604									
21	5.98	3.0445	18.20611	9.2690	35.7604									
22	5.99	3.0918	18.51988	9.5592	35.8801									
23	5.98	3.1355	18.75029	9.8314	35.7604									
24	5.98	3.1781	19.00504	10.1003	35.7604									
25	6.01	3.2189	19.34559	10.3613	36.1201									
26	5.98	3.2581	19.48344	10.6152	35.7604									
∑	156.54	61.2728	368.4345	161.9218	942.5226

**Table 3:** Learning Rate for Blockwork Craft gangs Productivity.

S/N	**LN Man-hours**	**LN Cycle No**	**C**	**D**	**E**	**F**	**G**	**H**	**I**	**J**	**K**	**L**	**M**	**O**	**P**	**Q**
	Y	X	XY	X2	n∑XY	∑X∑Y	n∑X2	(∑x)2	E-F	G-H	β=I/J	Ẏ	Ẋ	Βẋ	α=Ẏ-Βẋ	S=2b*100
1	6.1000	–	–	–	9,579.2959	9,591.6441	4,209.9665	3,754.3560	−12.3482	455.6105	**−0.0271**	6.0208	2.3566	−0.0639	**6.0846**	
2	6.0400	0.6931	4.1863	0.4804												
3	6.0300	1.1098	6.6921	1.2317												
4	6.0100	1.3862	8.3311	1.9216												**98.1389**
5	6.0100	1.6094	9.6725	2.5902												
6	6.0300	1.7917	10.8040	3.2102												
7	6.1200	1.9459	11.9089	3.7865												
8	6.0400	2.0794	12.5596	4.3239												
9	6.0500	2.1972	13.2931	4.8277												
10	6.0200	2.3025	13.8611	5.3015												
11	6.0400	2.3978	14.4827	5.7494												
12	6.0300	2.4849	14.9839	6.1747												
13	6.0400	2.5649	15.4920	6.5787												
14	6.0400	2.6390	15.9396	6.9643												
15	6.0000	2.7080	16.2480	7.3333												
16	6.0100	2.7725	16.6627	7.6868												
17	6.0400	2.8332	17.1125	8.0270												
18	5.9800	2.8903	17.2840	8.3538												
19	6.0100	2.9444	17.6958	8.6695												
20	5.9800	2.9957	17.9143	8.9742												
21	5.9800	3.0445	18.2061	9.2690												
22	5.9900	3.0918	18.5199	9.5592												
23	5.9800	3.1355	18.7503	9.8314												
24	5.9800	3.1781	19.0050	10.1003												
25	6.0100	3.2189	19.3456	10.3613												
26	5.9800	3.2581	19.4834	10.6152												
**∑**	156.5400	61.2728	368.4345	161.9218												

Secondly, the data set presented in Table 1, Table 2, Table 3 and Fig. 4 was derived from the observation study. A standard observation sheet and a stopwatch was used in recording the observed time for the craft gang's block laying operation in a working day. The data were collected daily to determine the variation in output for a total number of Twenty-six (26) observations from 7:00 a.m. to 6:00 p.m. daily.

## 2 Experimental design, materials and methods

The experimental data collection strategies used in this study is standard observation method and semi-structure Interviews.

### 2.1 Constraints influencing blockwork craft gangs productivity

The data presented in the interview were analysed via content analysis. Computer-assisted content analysis via NVivo 11 pro software was also used to aid the analysis. The participants interviewed were allocated a distinctive set of numbers. The reason for this numbers was for data coding system in order to determine the participant interviewed in the project in the analysis phase of the research. The number begins with the participant given as P, followed by the participant assigned number. For instance, if a participant is allocated with number P01, it means that P is the participant interviewed and was given the number 01.

The experimental design data on the project-specific constraints present the constraint with the highest response and rank. Fig. 1 shows the human/ motivation top project-specific constraints the craft gang's needed to respond to, in order to optimise their productivity.

Fig. 2 shows the top Project management constraints the craft gangs needed to respond to, in order to optimise their productivity.

Fig. 3 shows the top environmental, health and safety constraints the craft gang's needed to respond to, in order to optimise their productivity.

### 2.2 Influence of onsite learning on blockwork craft gang's productivity

Table 1 shows the data on regression correlation coefficients between blockwork inputs and cycle numbers at 5% significance level. The significance of the correlation coefficients was to determine the relationship between the data and the linear regression model. The coefficients were determined by substituting the linear regression model equation:

(1) Y=α+βX

The regression equation, α and β indicates the intercept and the slope of the linear regression model. The slope and the intercept are estimated thus;

(2) $$\beta =\left(n\sum \mathrm{xy}\;-\;\sum x\sum y\right)/\left(n\sum x^{2}\;-\;{\left(\sum x\right)}^{2}\right)$$

(3) $$\alpha =\overset{-}{Y}-\beta \overset{.}{X}$$

where *Y*, is the man-hours and *X*, is the Cycle Numbers.

In Table 2, *α*=6.08, *β*=−0.03, *ϒ*=−0.64 as presented in Table 1. Where α is the intercept given by the standard linear equation, β is the slope of the linear curve, *ϒ* is the correlation coefficient of the observed gangs. Hence, the general regression model for the observed blockwork craft gang's is given below as:

Υ=6.08−0.03X

That is, Man hours=6.08–0.03 cycle numbers.

The unit straight-line learning curve model was used to determine the role onsite learning play in the blockwork craft gangs learning productivity. The straight-line unit model is expressed as a power function [1], [2], [3]. The mathematical expressions underlying the logarithmic straight-line learning curve are:

(4) $$Y=T_{I}\times {\left(x\right)}^{b}$$

where *Y*=cost, man-hours, or time required to perform the repeated unit; *T*_1_=cost, man-hours, or time necessary to perform the first unit; *x*=cycle number of the unit; and b represents the slope of the logarithmic curve, which is calculated as:

(5) $$b=\frac{\mathrm{InS}}{\ln2}$$

where *S*=learning rate, which is defined as the percentage reduction in the unit input, i.e., cost, man-hours, or time, as a result of doubling the number of units completed. Eq. (5) can be rewritten as:

(6) $$S=(2^{b})*100$$

Fig. 4 and Table 3 presents data on the relationship between craft gang's blockwork and the cycle numbers. We also presented the learning rate (*S*), expressed as a percentage, this was determined by substituting the slope (b), that is −0.06, into the learning rate equation as follows: *S*=(2^−0.03^)×100.
